# Prevalence and Implications of Contamination in Public Genomic Resources: A Case Study of 43 Reference Arthropod Assemblies

**DOI:** 10.1534/g3.119.400758

**Published:** 2019-12-20

**Authors:** Clementine M. Francois, Faustine Durand, Emeric Figuet, Nicolas Galtier

**Affiliations:** UMR 5554, Institut des Sciences de l’Evolution; CNRS, University of Montpellier, IRD, EPHE, Montpellier, France

**Keywords:** contaminant sequences, horizontal gene transfer, automated detection pipeline, curation of genomic databases

## Abstract

Thanks to huge advances in sequencing technologies, genomic resources are increasingly being generated and shared by the scientific community. The quality of such public resources are therefore of critical importance. Errors due to contamination are particularly worrying; they are widespread, propagate across databases, and can compromise downstream analyses, especially the detection of horizontally-transferred sequences. However we still lack consistent and comprehensive assessments of contamination prevalence in public genomic data. Here we applied a standardized procedure for foreign sequence annotation to 43 published arthropod genomes from the widely used Ensembl Metazoa database. This method combines information on sequence similarity and synteny to identify contaminant and putative horizontally-transferred sequences in any genome assembly, provided that an adequate reference database is available. We uncovered considerable heterogeneity in quality among arthropod assemblies, some being devoid of contaminant sequences, whereas others included hundreds of contaminant genes. Contaminants far outnumbered horizontally-transferred genes and were a major confounder of their detection, quantification and analysis. We strongly recommend that automated standardized decontamination procedures be systematically embedded into the submission process to genomic databases.

Scientists typically re-use sequence data generated by others, and are therefore dependent on the reliability of the available genomic resources. For this reason, the problem of public data quality in molecular biology has long been identified as a crucial issue (Lamperti *et al.* 1992; Mistry *et al.* 1993; Binns 1993). The problem is even more acute nowadays with the advent of high-throughput sequencing technologies, when most datasets generated in genomic research are simply not amenable to manual curation by humans. This brings a new challenge to current methodologies in genomic sciences, namely, the development of automated approaches to the detection and processing of errors (*e.g.*, Andorf *et al.* 2007; Schmieder and Edwards 2011; Parks *et al.* 2015; Delmont and Eren 2016; Drăgan *et al.* 2016; Tennessen *et al.* 2016; Laetsch and Blaxter 2017; Lee *et al.* 2017).

Data quality issues in genome sequences include sequencing errors, assembly errors and contamination, among other things. Errors due to contamination are particularly worrying for several reasons. First, they can lead to serious mis-interpretations of the data, as illustrated by recent, spectacular examples. Potential problems include mis-characterization of gene content and related metabolic functions (*e.g.*, Koutsovoulos *et al.* 2016; Breitwieser *et al.* 2019), improper inference of evolutionary events (*e.g.*, Laurin-Lemay *et al.* 2012; Simion *et al.* 2018), and biases in genotype calling and population genomic analyses (*e.g.*, Ballenghien *et al.* 2017; Wilson *et al.* 2018). Second, contamination is suspected to be widespread. It occurs naturally in most sequencing projects due to foreign DNA initially present in the raw biological material (*e.g.*, symbionts, parasites, ingested food; Salzberg *et al.* 2005; Starcevic *et al.* 2008; Artamonova and Mushegian 2013; Driscoll *et al.* 2013; Martinson *et al.* 2014; Cornet *et al.* 2018), or entering the process in wet labs and sequencing centers (Longo *et al.* 2011; Salter *et al.* 2014; Wilson *et al.* 2018). Third, contamination errors easily propagate across databases in a self-reinforcing vicious circle. If a DNA sequence from species A is initially assigned to the wrong species B due to a contamination of B by A, it is likely to keep its incorrect status for a while, and may even be identified as a contamination of A by B when the genome of A is eventually sequenced (Merchant *et al.* 2014). Despite all the possible problems stemming from contamination in genomic resources, most studies addressing this issue so far have focused on one particular genome (*e.g.*, tardigrades) and/or one particular source of contaminants (*e.g.*, humans). Only two studies that we are aware of have consistently screened more than one genome assembly. Merchant *et al.* (2014) focused on the bovine genome but also applied their pipeline to eight randomly drawn draft genomes (five animals, two plants, one fungus), with contrasted results. Cornet *et al.* (2018) analyzed 440 genomes of Cyanobacteria and uncovered a substantial level of contamination in >5% of these. There is obviously a need for further assessment of the problem of contamination in publicly available genomic data.

Probably the research goal most sensitive to contamination is the detection of horizontally-transferred genes – nothing resembles a transferred sequence more than a contaminant does. Horizontal gene transfer (HGT) between species is a pervasive process in prokaryotes, which dramatically affects gene phylogenies and species ability to adapt to environmental changes (Ochman *et al.* 2000; Koonin 2016). Whether it substantially influences genome evolution also in large eukaryotes is a matter of debate (Andersson 2005; Boto 2014). A number of examples are documented (*e.g.*, Schönknecht *et al.* 2014), but a quantitative assessment of the prevalence of HGT in eukaryotes is difficult, and many HGT candidates were subsequently shown to result from contamination. Controversies over the confusion between HGT and contaminants have concerned the human genome (Willerslev *et al.* 2002; Salzberg 2017), the *Nematostella vectensis* sea anemone genome (Starcevic *et al.* 2008; Artamonova *et al.* 2015), and the *Hypsibius dujardini* tardigrade genome, among others. In *H. dujardini*, the initial estimate of 17% of genes being of foreign origin was revisited to 1% when contamination was properly accounted for (Hashimoto *et al.* 2016; Koutsovoulos *et al.* 2016).

A straightforward way to identify contamination in a newly sequenced genome is to compare the assembled sequences to existing databases using BLAST-like algorithms. If a sequence’s best match is assigned to a species that is phylogenetically distant from the target organism, then the sequence is annotated as a contaminant. There are several problems with this simple strategy. First, this does not allow one to distinguish contaminants from HGT. Second, this approach is entirely dependent on the correctness of the reference database. A best-BLAST-hit survey can only propagate, not correct, pre-existing taxonomic mis-assignments, as discussed above. Third, such an approach is also dependent on the completeness of the reference database, and on the phylogenetic position of the target organism. If the reference database is imbalanced and dominated by one or a few particular taxa (typically model organisms), then its power to properly discriminate genuine sequences from contaminants will be maximal for newly sequenced organisms closely related to the dominant taxa, and much lower for organisms distantly related to the dominant taxa.

Solutions to these problems exist, and include (i) considering multiple BLAST hits, not just the “best” one, (ii) using an appropriately balanced reference database, (iii) incorporating information on synteny (*i.e.*, physical co-localization of loci on the same scaffold), and ultimately phylogeny, in addition to sequence similarity. Here we collated these ideas in an integrated framework aiming at properly quantifying the prevalence of contamination in genomic data based on reliable, existing tools. We applied this pipeline to 43 published genomes of arthropods distributed in the Ensembl database. We report that data quality is highly heterogeneous across species in this widely used database, some genomes being heavily affected by contamination. Our results also show that a careful annotation of contaminant sequences is mandatory in any subsequent attempt to detect HGT.

## Materials & Methods

### Foreign sequence annotation

**Figure 1:**
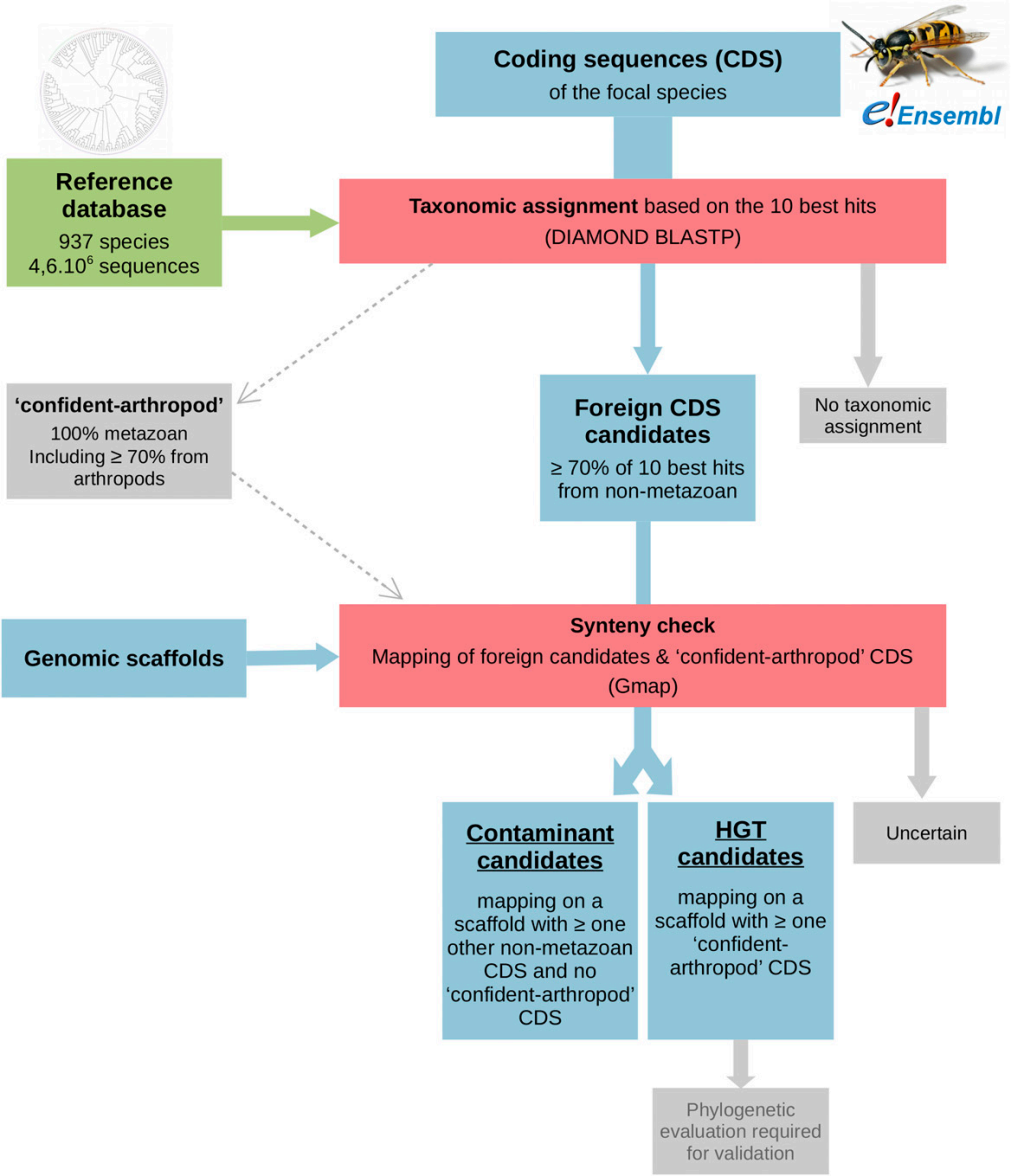
A simplified flow diagram of the pipeline developed for this study. Each species assembly is evaluated independently through this pipeline, which requires the set of coding sequences (CDS) as well as the genomic scaffolds of each species, and an appropriate reference database. In this diagram, boxes referring to ‘data’, ‘reference database’ and ‘tools’ are colored in blue, green, and red, respectively. See the main text for detailed explanations.

We developped a dedicated pipeline for the simultaneous detection of contaminants and HGT candidates in published genome assemblies. This pipeline was optimized and benchmarked in arthropods, but can be applied to any other taxa, provided that an adequate reference database is available. The outline of the pipeline is presented in Figure 1. It takes as input a genome assembly and a set of predicted coding sequences (CDS). It returns a set of CDS annotations with the following categories: genuine arthropod gene, HGT candidate, contaminant candidate, orphan gene, uncertain. Five non-metazoan taxonomic groups are considered as potential sources of contaminants and HGT: eubacteria, archaea, fungi, viridiplantae and ‘protists’. Each investigated genome is processed independently and without any *a priori* on the source(s) of contamination. As discussed below, the power of this pipeline to detect foreign sequences depends on the level of fragmentation of the considered assembly.

The first step of the pipeline is a preliminary taxonomic assignment of CDS based on sequence similarity. Using DIAMOND BLASTP (v0.8.22, “more-sensitive” mode, otherwise default parameters; Buchfink *et al.* 2015), each CDS was blasted against a custom protein reference database (see below). Hits with identity greater than 40%, alignment length greater than 75 amino-acids and E-value lower than 10^−10^ were retained. A minimum of two such hits to two different species was required for taxonomic assignment. CDS not matching this criterion were regarded as orphan genes (‘no reliable taxonomic assignment’) and not considered further. For each CDS, the 10 hits with the smallest E-values were considered – or less if less than 10 hits had an e-value below 10^−10^. A CDS was assigned to a given taxonomic group (*i.e.*, eubacteria; archaea; viridiplantae; fungi; protists) if at least 70% of its best hits fell within this group. These were called “foreign CDS candidates”. In addition, a CDS was assigned to the “confident-arthropod” group if 100% of its best hits were to a species of Metazoa, among which at least 70% to a species of Arthropoda. Finally, a CDS was assigned to “other metazoa” if at least 70% of its best hits were to species of non-arthropod metazoa, and none to a species of arthropods. CDS not matching any of these criteria were considered taxonomically unassigned. Using the 10 best hits instead of just the best one provides a robust way to account for potential contaminations and other sources of taxonomic mis-assignment in the reference database. The 70% threshold was empirically determined as providing a reasonable trade-off between sensitivity and specificity.

The second step of the pipeline is a test of synteny. All foreign CDS candidates as well as the “confident-arthropod” CDS were mapped onto the species genomic scaffolds using GMAP (v2017-04-24; Wu and Watanabe 2005) with the option “-npaths=0”. To account for variable fragmentation of genome assemblies (*i.e.*, N50), we allow for “chimeric alignments” (*i.e.*, CDS whose 5 and 3 ends map to different scaffolds). We required a minimum alignment length of 100bp and a minimum identity of 95%. A foreign CDS candidate was considered as a HGT candidate if it was physically linked to (*i.e.*, mapped to the same scaffold as) at least one “confident-arthropod” CDS. A foreign CDS candidate was considered as a contaminant candidate if it mapped to a scaffold to which no “arthropod-confident” CDS mapped, and at least another non-metazoa CDS mapped. A foreign CDS candidate was considered as “uncertain” if it did not reliably map to any scaffold or if it was the only CDS to map to a given scaffold. When present, the “confident-arthropod” tag was propagated across all scaffolds linked by chimeric alignments. This synteny-based step can also be performed at the contig scale in case of doubts regarding the scaffolding process; this should increase the proportion of foreign candidates classified as “uncertain”.

The corresponding script is available on GitHub (https://github.com/ClementineFrancois/Foreign-CDS-detection). The analysis of the 43 arthropod assemblies of this study took around 48 hr to run on 50 CPU.

### Evaluated genome assemblies

The 43 arthropod genomes available in Ensembl Metazoa (Release 37, as of October 2017; Kersey *et al.* 2017) were investigated using our dedicated pipeline. This included 36 insects, two crustaceans, four chelicerates and one myriapod (see Supplementary Table S1). For each species, the set of masked genomic scaffolds (“dna_rm.toplevel”) as well as the set of all predicted coding sequences (“cds.all”) were retrieved from Ensembl Metazoa. Depending on the species, the set of annotated CDS was either generated by Ensembl or imported from other reference databases relying on different annotation pipelines. Scaffolds shorter than 200 bp were discarded. The longest transcript was selected for each gene. Coding sequences shorter than 150 bp were discarded.

### Custom reference database

A custom protein reference database was built to cover all domains of life and included 937 species (4,622,809 sequences). The proteomes of 100 eukaryotic species were retrieved from Ensembl (Release 90; Zerbino *et al.* 2017) and Ensembl Metazoa (Release 37; Kersey *et al.* 2017). These included 40 metazoa (of which 20 arthropods), 20 fungi (of which 10 fungi known to infect arthropods), 20 Viridiplantae and 20 ’protists’. The proteomes of 837 prokaryotic species, of which 748 eubacteria and 89 archae, were retrieved from the Microbial Genome Database for Comparative Analysis (mbgd_2016-01; Uchiyama *et al.* 2014) selecting one species per genus. An additional 11 known symbionts of arthropods were subsequently included. Within each proteome, redundant sequences (>90% identity) were removed using CD-HIT (Fu *et al.* 2012). Information on the content of the custom reference database is provided in Supplementary Table S2.

### Validation of the contaminant candidates

In two species of interest, the tetranucleotide (4-mer) frequencies of candidate contaminant CDS were visually compared to those of “confident-arthropod” CDS using a Principal Components Analysis (PCA). PCA was performed in R using the ‘prcomp’ function and results were plotted using the ‘pca3d’ package.

### Validation of the HGT candidates

We took a phylogenetic approach to validate / invalidate HGT candidates in one species of interest, the pea aphid *Acyrthosiphon pisum*. All HGT candidates detected in the pea aphid assembly were clustered into families with Silix (v1.2; Miele *et al.* 2011), requiring a minimum of 60% of identity (default parameters otherwise). For each family, a protein alignment of the candidate HGT sequence(s) and its (their) 50 best BLAST hits in the custom reference database was generated with MAFFT (v7; Katoh and Standley 2013). Only BLAST hits with identity greater than 40%, alignment length greater than 75 amino-acids and E-value lower than 10^−10^ were considered. The alignements were cleaned using HMMcleaner (stringency parameter = 12). Phylogenetic trees were inferred using RAxML (v8.2; Stamatakis 2014) with the model ‘PROTGAMMALGX’ of amino-acid substitution and 100 bootstrap replicates. Phylogenetic trees were inspected by eye.

### Statistical analyses

According to the recommendations of Warton and Hui (2011), all proportion data were logit-transformed prior to statistical analyses, using the ‘car’ R package (Fox and Weisberg 2011). The normality of the residuals was checked for all models reported in this article. All data analyses were performed with R 3.4 software (R Core Team 2018) using the vegan (Oksanen *et al.* 2018) and seqinr (Charif and Lobry 2007) packages.

### Data availability

This study is based on publicly available data from the Ensembl database (the accession numbers are listed in Table S1).

Table S1 describes the genomic features of the 43 arthropod species from EnsemblMetazoa investigated in this study. Table S2 details the composition of the custom reference database. Table S3 describes the categorization of all CDS in the 43 arthropod genomes. Table S4 indicates the inferred function and potential donor for the six validated HGT families in the pea aphid assembly.

Figure S1 shows the correlation between the log-transformed N50 of each genome assembly and the percentage of foreign CDS candidates initially identified in the 1^st^ similarity-based step of the pipeline which were subsequently considered as uncertain in the 2^nd^ synteny-based step. Figure S2 shows the number of contaminant and HGT candidates detected in each of the 43 arthropod genomes, according to the assembly N50. Figure S3 displays the Principal Components Analysis of CDS tetranucleotide frequencies in the pea aphid and bumblebee assemblies. Figure S4 shows the distribution of the number of contaminant CDS per contaminant scaffold. Figure S5 contains the RAxML phylogenies inferred for the six validated HGT families in the pea aphid assembly.

Bioinformatic scripts are available on GitHub (https://github.com/ClementineFrancois/Foreign-CDS-detection). Supplemental material available at figshare: https://doi.org/10.25387/g3.9890894.

## Results & Discussion

### Overview of the 43 arthropod genomes: contamination

**Figure 2:**
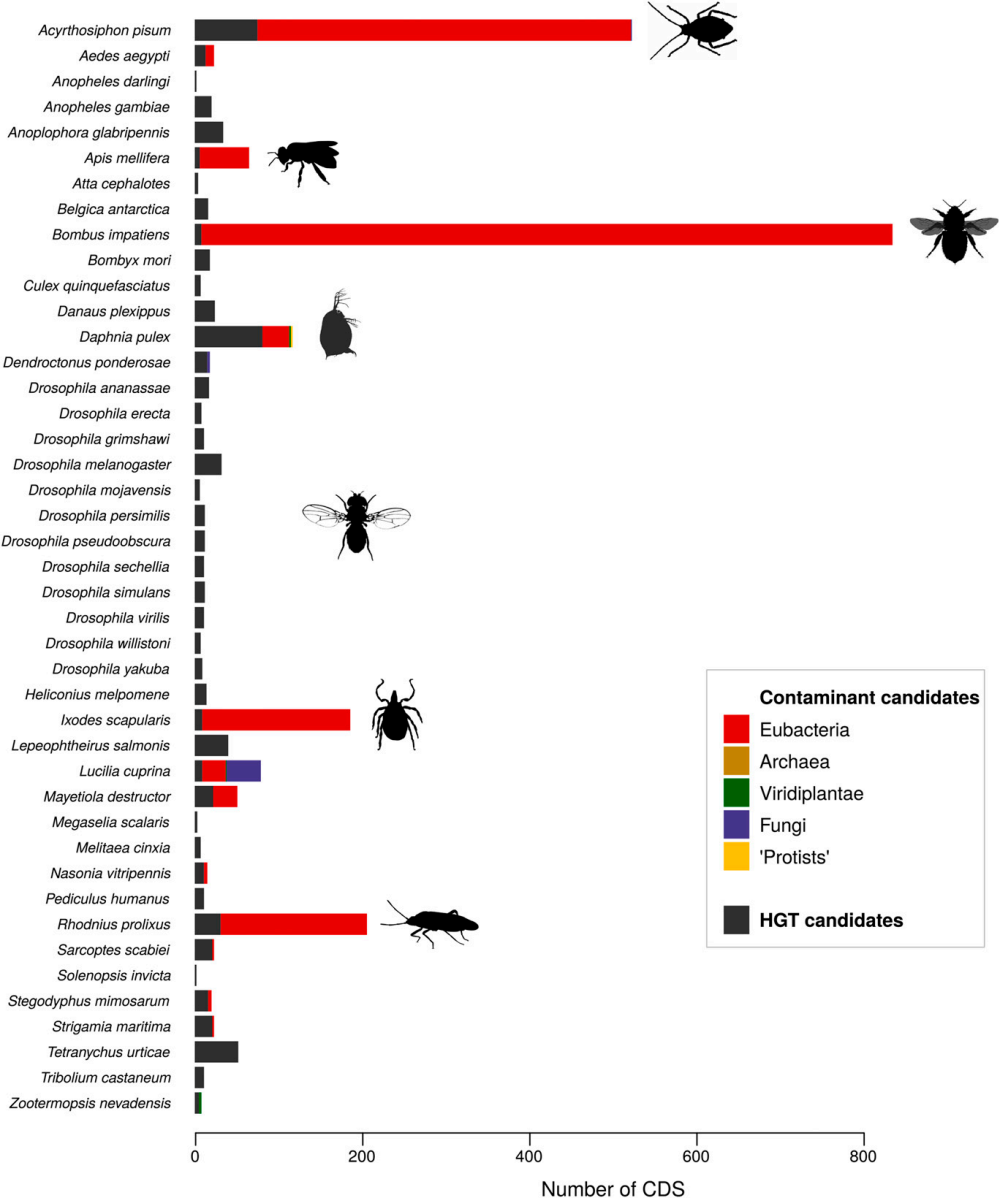
Prevalence of contaminant and HGT candidates in the 43 arthropod genomes. Contaminant CDS are classified according to their taxonomic group (*i.e.*, originating from eubacteria, archaea, viridiplantae, fungi and ‘protists’). Images courtesy of PhyloPic.

We applied our newly introduced contamination/HGT annotation pipeline to 43 assemblies from Ensembl Metazoa. Detailed results are displayed in Figure 2 and Suppl. Tables S1 & S3. Out of 43 arthropod assemblies, 28 were completely devoid of non-metaozan contamination (including the 12 *Drosophila* species), while 4 of them contained more than 150 contaminant CDS. The number of predicted contaminant CDS per assembly ranged from 0 to 827 among species, representing 0–5% of all CDS, and 0–8% of the CDS for which a taxonomy assignment was possible – which is probably the most meaningful measure of the prevalence of contamination (Suppl. Table S1). The most contaminated assemblies were those of the bumblebee (*Bombus impatiens*) and the pea aphid (*Acyrthosiphon pisum*).

The number of contaminant scaffolds (*i.e.*, containing at least two contaminant CDS and no genuine arthropod CDS) varied from 0 to 202 across assemblies (Suppl. Table S1). The contaminant CDS were either scattered across many small scaffolds (*e.g.*, 448 contaminant CDS distributed across 202 scaffolds in the pea aphid) or carried by just a few long contaminant scaffolds (*e.g.*, 827 contaminant CDS in 30 scaffolds in the bumblebee). The size of contaminant scaffolds ranged from 602 bp (in the barley midge *Mayetiola destructor*) to 793,321 bp (in the deer tick *Ixodes scapularis*), and their cumulative length represents up to 2,497,466 bp in the pea aphid *Acyrthosiphon pisum*.

As an evaluation of the reliability of our results, we tested the taxonomic consistency of the contaminant scaffolds detected in our analyses. Indeed, all CDS encoded on a given contaminant scaffold are expected to derive from the same organism, thus to be assigned to the same non-metazoan group (*e.g.*, eubacteria). Out of 408 detected contaminant scaffolds, only one was taxonomically inconsistent. This 20kb scaffold from the *Lucilia cuprina* (blowfly) assembly encoded one eubacterial and two fungal CDS. It could be a chimera between two contaminant sequences.

The great majority of detected contaminations originated from eubacteria (1,796 out of 1,849 contaminant candidates for the 43 species), except in blowfly *Lucilia cuprina* which was mostly contaminated by fungal sequences (41 CDS; Figure 2 and Suppl. Table S3). The fact that no archaeal contamination was detected in any assembly (Suppl. Table S3) might at least in part reflect a taxonomic gap in the public reference databases. This problem has already been evidenced from the study of mammalian gut microbiota (Raymann *et al.* 2017) and likely impacts all database-dependent studies.

In summary, out of 43 published genome assemblies, 15 (*i.e.*, 35%) presented at least some traces of non-metazoan contamination, including four which were substantially contaminated. These figures are likely an underestimation of the actual prevalence of contamination because of the limitation due to incompleteness of reference genomic databases, as discussed above. Moreover, the overall prevalence of contamination is expected to be even higher as we did not consider metazoan contaminants. Yet contamination from wet lab technicians as well as from model organisms extensively used in research facilities (*e.g.*, mouse, zebrafish, …) is likely to occur in any sequencing project. Our results are consistent with recent analyses which uncovered similar level of contamination in published genome assemblies (*e.g.*, Borner and Burmester 2017). In particular, Bemm *et al.* (BioRxiv: https://doi.org/10.1101/122309) reported from 0 to ca. 5% of bacterial contamination in Ensembl Metazoa genome and identified the bumblebee *Bombus impatiens* as one of the most highly contaminated assemblies. In addition, our analyses focused on CDS, which are among the most conserved and easy to annotate sequences of a genome, *i.e.*, probably most easily filtered for contamination by assembly pipelines. Therefore the situation regarding contamination is probably even worse as far as non-coding sequences are concerned.

### Overview of the 43 arthropod genomes: HGT candidates

In this study, potential HGT candidates were detected at very low level in all genome assemblies. Across the 43 investigated species, the number of CDS suspected to derive from an HGT event ranged from 2 to 81 per assembly, with a median of 12 HGT candidates (Figure 2, Suppl. Tables S1 and S3). The HGT candidates represented up to 1.25% of the taxonomically-assigned CDS for a given species (in the spider mite *Tetranychus urticae*). These HGT candidates have yet to be validated. These results are consistent with several recent studies on this species which evidenced an unexpectedly high level of putative HGT from bacteria and fungi (Grbić *et al.* 2011; Ahn *et al.* 2014; Altincicek *et al.* 2012; Wybouw *et al.* 2012). The proportion of HGT candidates was rather variable among species, and substantially lower than 1% in a large majority of species (median = 0.14%). Around a third of all detected HGT candidates likely originated from eubacterial donors, and another third from viridiplantae ones (respectively 286 and 295 candidates out of 756; Suppl. Table S3). Similarly to contaminant candidates, very few putative archaeal HGT were detected.

These preliminary results should be considered with a high degree of caution as HGT candidates have not been validated through a phylogenetic approach or an experimental confirmation via PCR or re-sequencing, so the prevalence of HGT in arthropod genomes is likely over-estimated. Still these preliminary results can be compared to previous HGT studies on Metazoa. In a review including 8 metazoan species (Schönknecht *et al.* 2014), the number of phylogenetically-supported HGT ranged from 12 to 198 genes across species (with the repeatedly documented exception of the bdelloid rotifers containing 2,700 HGT; see Nowell *et al.* 2018). In another study, Crisp and colleagues (2015) analyzed 26 metazoan genomes and identified from 2 to 100 HGT across species. Both studies evidenced the same order of HGT prevalence in metazoans as we preliminary did on arthropods.

### Influence of the fragmentation level of the assembly

Our ability to detect contaminants and HGT decreases with the fragmentation of genome assembly. Indeed, highly fragmented assemblies contain many small scaffolds which are more likely to encode a single CDS. If detected as suspicious in the first step of the pipeline, such CDS (*i.e.*, alone on their scaffold) would then be considered as uncertain in the second synteny-based step, thus decreasing the power of our pipeline.

The N50 was highly variable across the 43 arthropod assemblies, ranging from 2.3 kb (in the fly *Megaselia scalaris*) to 41.5 Mb (in the mosquito *Anopheles gambiae*), with a median at 742 kb (Suppl Table S1). We found a negative correlation between genome assembly N50 and the percentage of foreign CDS candidates classified as “uncertain” at the second step of the pipeline (linear model, p-value = 0.0002, R^2^ = 0.2854; Suppl. Figure S1). This indicates that the actual prevalence of contamination was underestimated in our study. Of note, despite the decreased power to detect contaminants and HGT in fragmented assemblies, our pipeline identified high amounts of putative contaminants and HGT in some low-N50 genomes (Suppl. Figure S2). As a matter of fact, the highest contamination levels were identified in low-N50 assemblies (Bombus impatiens, N50 = 1.3 Mb; Acyrthosiphon pisum, N50 = 431 kb).

### Detailed investigation in three species

Further analyses were performed in three species of interest: the pea aphid (*Acyrthosiphon pisum*), the bumblebee (*Bombus impatiens*) and the fruit fly (*Drosophila ananassae*). We assessed the reliability of our sets of contaminant / HGT candidates and discussed their origin through the analyses of their tetranucleotide content, across-scaffold distribution, and phylogeny.

#### The case of the pea aphid (Acyrthosiphon pisum):

The genome assembly of the pea aphid showed one of the highest number of predicted contaminant and horizontally-transferred CDS (Suppl. Table S1). We thus investigated in more details the sets of contaminant and HGT candidates.

The phylogenetic signal repeatedly described in tetranucleotide (4-mer) frequencies of CDS means that such frequency patterns convey information about the evolutionary history of the sequence (Pride *et al.* 2003; Teeling *et al.* 2004; Dick *et al.* 2009) and should theoretically enable to discriminate between contaminant and arthropod sequences, similarly to the rationale behind the Blobtools suite (which considers the scaffold %GC; Laetsch and Blaxter 2017) or the algorithm CONCOCT (for the automated binning of metagenomic contigs; Alneberg *et al.* 2014). The 4-mer frequencies of the contaminant candidates identified in the pea aphid assembly, as well as those of the ‘confident-arthropod’ CDS, were visualized using a PCA (plotting the three principal components). Almost all contaminant candidates fall outside of the cluster of resident arthropod genes (Suppl. Figure S3a), supporting the reliability of the set of contaminants identified in the pea aphid assembly.

In this assembly, the 448 predicted contaminant CDS derived from 202 scaffolds. Contaminant CDS were scattered across many small scaffolds harboring only a few CDS each, a pattern similar to most of the screeened assemblies (Suppl. Figure S4). 99.5% of the contaminant scaffolds (201 out of 202) were from eubacterial origin. An examination of the taxonomy of BLAST hits indicated that a vast majority of contaminant sequences originated from donors of the order Enterobacterales, and showed closest matches to species of the families Enterobacteriaceae and Erwiniaceae. Interestingly, these two families contain several well-described bacterial symbionts of aphids, such as the obligate endosymbiont *Buchnera aphidicola* or the facultative symbionts *Hamiltonella defensa* and *Serratia symbiotica* (Oliver *et al.* 2010). However, none of the detected contaminant CDS blasted reliably on the genome of *Buchnera aphidicola* nor on the genomes of common aphid secondary symbionts (*Hamiltonella defensa*, *Serratia symbiotica*, *Spiroplasma*, *Cardinium* and *Rickettsia*), although these species were represented in our reference database. Symbiont-derived sequences were likely present in the raw dataset and subsequently removed from the assembly, which is a common procedure in sequencing projects (*e.g.*, see International Aphid Genomics Consortium 2010). This targeted cleaning approach can only be applied to well-known symbionts of the focal organism. The remaining contaminant sequences might thus correspond to less studied aphid symbionts, such as species of the genera *Pantoea or Erwinia*, which showed strong BLAST matches with contaminant CDS and have been identified as aphid gut symbionts (Harada *et al.* 1997; Gauthier *et al.* 2015).

The 75 HGT candidates detected in the pea aphid assembly clustered in 70 gene families, from which gene phylogenies were constructed. The 70 trees were inspected by eye, and only six of them were considered as reliably supporting an instance of HGT (Suppl. Figure S5). The other 64 trees were disregarded mainly because the terminal branch leading to the putatively-transferred sequence was too long for a reliable phylogenetic placement. Five HGT likely originated from eubacterial donors (Suppl. Table S4), including a transposase gene. The remaining HGT concerned four CDS, which were likely acquired from a fungus (Suppl. Figure S5, Suppl. Table S4). The functional annotations of the best BLASTP hits (NR database) suggest that the horizontally-transferred genes of fungal origin encode a phytoene desaturase, an enzyme involved in carotenoid biosynthesis. This result is congruent with previous studies in aphids wich indicated that the phytoene desaturase gene had undergone several duplication events after its transfer from a fungal donor (Nováková and Moran 2011). This HGT event seems to be ancient and shared with the red spider mite T*etranychus urticae* (Altincicek *et al.* 2011; Grbić *et al.* 2011), which is consistent with the phylogenetic tree we inferred for this gene family (*cf*. Suppl. Figure S5). Carotenoid pigments can confer many essential benefits (*e.g.*, protection from oxidative damage, light detection, photoprotection, signaling) and are acquired by most animals from their diet. HGT events enabling an organism to *de novo* synthetise carotenoids could confer a substantial adaptive advantage to the recipient species (Bryon *et al.* 2017).

Of note, only a minority of the suspected HGT (six out of 70) were confirmed via our phylogenetic analysis. This confirms that evidence solely based on sequence similarity are not sufficient to demonstrate the existence of an HGT event, far from it. A phylogeny-based validation is required. For example the controversy on human genome demonstrated that most, if not all, putative horizontally-transferred sequences initially identified through a BLAST approach, actually originated from classical vertical descent (Stanhope *et al.* 2001).

#### The case of the bumblebee (Bombus impatiens):

The genome assembly of the bumblebee represents a particularly striking example of host genome contamination by symbiont sequences. In this assembly, the 827 predicted contaminant CDS derived from only 30 scaffolds.

Using the same approach as described above in the pea aphid, the 4-mer frequencies of contaminant candidates and ‘confident-arthropod’ sequences were visualized using a PCA, which clearly separated the two sets of CDS (Suppl. Figure S3b). This pattern supported the reliability of the set of contaminants identified in the bumblebee assembly.

The 827 contaminant CDS were concentrated in only 30 contaminant scaffolds harboring up to 108 CDS each, a pattern strikingly different from the other assemblies we analyzed (Suppl. Figure S4). All contaminant sequences were of eubacterial origin, and ca. 97% (799 out of 827) consistently showed high sequence similarity with two species of the Orbaceae family present in our reference database, namely *Gilliamella apicola* and *Frischella perrara*. These 799 Orbaceae CDS correspond to just 25 contaminant scaffolds, the lengths of which sum up to 2,157,077 bp. *Gilliamella apicola* is known to be a gut symbiont of bumblebees and its genome size is ∼2.2 Mb (Kwong and Moran 2013), suggesting at first sight that the whole genome of this species could be included in the bumblebee assembly. However, Martinson *et al.* (2014) described a new bumblebee gut symbiont sequenced concurrently with the genome of its host. This symbiont, *Candidatus* Schmidhempelia bombi, is another good candidate as it was not present in our reference database, has a genome size of at least 2 Mb, and shares significant sequence similarity with *Frischella perrara* and *Gilliamella apicola*. All contaminant CDS were blasted against the three assemblies available in NCBI (*Candidatus* Schmidhempelia bombi str. Bimp; *Gilliamella apicola* str. WkB30; *Frischella perrara* str. PEB0191). 769 CDS out of 827 showed 100% nucleotide similarity with sequences of *Candidatus* Schmidhempelia bombi. The maximum sequence similarity with *Frischella perrara* and *Gilliamella apicola* were 95% et 87%, respectively. We conclude that almost the entire genome of *Candidatus* Schmidhempelia bombi is present in the bumblebee assembly distributed by Ensembl Metazoa, although this symbiont was described and its genome sequence published in 2014 (Martinson *et al.* 2014).

The bumblebee assembly is therefore a textbook example of a complete symbiont genome accidentally sequenced alongside the focal organism and mistakenly incorporated into the primary assembly (Sadd *et al.* 2015). As of today, while both NCBI and the European Nucleotide Archive have twice updated the bumblebee assembly since March 2018 (exclusion of bacterial sequences; BIMP_2.2, GCA_000188095.4), EnsemblMetazoa is still distributing the first version of the assembly (BIMP_2.0) which includes the endosymbiont sequences, with obvious implications regarding downstream analyses.

#### The case of Drosophila ananassae:

We focused on *Drosophila ananassae* because several studies demonstrated widespread HGT from *Wolbachia* into the genome of this species (Hotopp *et al.* 2007; Klasson *et al.* 2014). However, only three eubacterial HGT candidates were detected by our pipeline, even though four *Wolbachia* strains were present in our reference database. Besides, none of these HGT candidates showed any good match with *Wolbachia* sequences when blasted against NR NCBI. This discrepancy could have been explained if these HGT occurred a long time ago, causing horizontally-transferred sequences to degenerate beyond the point where they would be recognized as CDS, and thus would not have been screened in our pipeline. However, at least 28 of these *Wolbachia* horizontally-transferred sequences seem to be expressed at low abundance in *D. ananassae* (Hotopp *et al.* 2007), suggesting that they are not too degenerate to be transcribed. Another explanation would be an excessive cleaning of *Drosophila ananassae* assembly causing all foreign (HGT and contaminant) sequences to be systematically removed, regardless of their physical integration into the fly genome. This would explain why none of the previously described HGT was detected. This hypothesis is supported by the fact that no contaminant sequence was detected in any of the 12 *Drosophila* assemblies (Suppl. Table S1). A last hypothesis would be that horizontally-transferred sequences are still functional and present on the genomic scaffolds, but were somehow filtered out during the annotation step (prediction of CDS; imported from FlyBase for all Drosophila species) and thus were not screened by our pipeline. This hypothesis is supported by the fact that 239 proteins of the *Wolbachia* reference assembly (ASM367136v1, NCBI) showed good BLASTX matches with the genomic scaffolds of *Drosophila ananassae* (E-value < 10^−30^). It should be noted that Ensembl has updated the gene set of *D. ananassae* since our analyses, adding thousands of CDS (release dana_r1.05 from FlyBase), including some with high similarity to Wolbachia sequences.

This example illustrates the potential downstream impacts of the cleaning and annotation procedures implemented in genome sequencing projects, which can result in *bona fide* genes of interest being discarded, and therefore taken away from the genomic databases and literature. Moreover, the lack of specific documentation on the procedures implemented for each assembly makes the (frequent) successive versions changes hardly tractable for the users, although these can have substantial impact on the distributed genomic data.

## Conclusions

Identifying genes of foreign origin in a genome is a goal of major biological interest, which is required to properly account for the problem of contamination in published genome assemblies. Applying a consistent, automated, reproducible foreign sequence annotation pipeline, we revealed considerable heterogeneity among arthropod genomes from the Ensembl Metazoa database in terms of prevalence of contaminants. Of the 43 arthropod assemblies we analyzed, 28 were completely devoid of contaminant sequences (including the 12 *Drosophila* species), 11 included a few, while four of them were heavily affected (> 150 contaminant CDS). The highest level of contamination was detected in the bumblebee assembly which contained 827 contaminant CDS likely originating from a single endosymbiont. This disparity between entries of a single, widely used database is worth noting, beyond the heterogeneity of annotation procedures among genome assemblies. Some of the Ensembl Metazoa assemblies were “cleaned” to the point that previously documented HGT have been removed, whereas others included hundreds of contaminant genes. Most of the detected foreign sequences proved to be contaminants, while very few HGT were confirmed. Therefore any analysis of HGT solely based on existing gene annotations would presumably yield results of little, if any, biological relevance. Contamination is in large part unavoidable and a major confounder of all downstream genomic analyses. While researchers should be accountable for the cleaning of their NGS datasets prior to distribution, there is inevitably some heterogeneity among labs and consortiums in terms of procedures and scientific goals. Thus we recommend that reproducible decontamination procedures (*e.g.*, Tennessen *et al.* 2016; Laetsch and Blaxter 2017; this study) be systematically embedded into the submission process to genomic databases.
